# Valorization of Lavender Agro-Waste into Functional Carbon Materials via Carbonization and Zn^2+^ Modification

**DOI:** 10.3390/molecules31030540

**Published:** 2026-02-03

**Authors:** Ognyan Sandov, Lyudmila Krasteva, Iliyana Naydenova, Ivan Kralov, Georgi Todorov, Tsvetelina Petrova

**Affiliations:** 1 Technical College—Sofia, Technical University of Sofia, 1000 Sofia, Bulgaria; o.sandov@tu-sofia.bg (O.S.); tzvetelina.petrova@tu-sofia.bg (T.P.); 2 Faculty of Industrial Technology, Technical University of Sofia, 1756 Sofia, Bulgaria; 3 Faculty of Transport, Technical University of Sofia, 1756 Sofia, Bulgaria

**Keywords:** lavender residue, biochar, sustainable materials, zinc modification, Zn-modified biochar

## Abstract

This paper proposes a valorization approach for solid lavender residue, a by-product of the essential oil industry. The biomass residue was carbonized at atmospheric pressure and two temperatures (450 °C and 650 °C), followed by solvothermal modification with zinc ions (Zn^2+^, 3 and 5 mmol). The effects of temperature and Zn^2+^ incorporation on the elemental composition and morphology of the resulting biochar were examined using X-ray Fluorescence (XRF), Fourier Transform Infrared (FTIR) spectroscopy, and Scanning Electron Microscopy/Energy-Dispersive X-ray Spectroscopy (SEM/EDS) analyses. The applied Zn^2+^ modification was effective at both concentrations for the biochar obtained at both carbonization temperatures. However, a more uniform metal ion distribution was observed at 3 mmol, while at 5 mmol, a partial particle agglomeration occurred. Progressive degradation of the O–H, C=O, and C–O groups with increasing temperature and the presence of Zn–O-related interactions was observed. The results demonstrated consistent and reproducible trends, suggesting that controlled carbonization combined with Zn^2+^ incorporation can convert lavender residues into modified carbonaceous materials.

## 1. Introduction

*Lavandula angustifolia* is of great importance to the essential oil industry worldwide. In the European Union (EU), the plant is cultivated primarily in France, Bulgaria, and Spain [1,2]. Recent work presented by Khiri et al. [3] reported an annual production of the global medicinal plant industry of approximately 20 million tons. A study by Crisan et al. [4] estimated that the essential oils derived from species and hybrids within the genus Lavandula collectively reach an annual production of approximately 1500 tons worldwide. Khatri et al. [5] commented that, besides the extensive research on lavender essential oil and liquid by-products, the remaining solid residues are often underexploited. Chilev et al. [6] estimated the annual generation of at least 20,000 tons of lavender solid residue from steam distilleries in Bulgaria. A significant fraction of this biomass remains unused. Despite its valuable lignocellulosic constituents, it is typically treated as solid waste, whose uncontrolled disposal might cause environmental concerns [2].

Increasing research efforts are being directed toward the valorization of agro-forest residues for applications such as water purification [7,8,9], soil remediation [10,11,12], catalytic materials, functional carbon-based products [13,14,15,16,17,18,19,20], and many others. Thermo-chemical conversion of agro-industrial residues into biochar is among the well-accepted valorization pathways [21] and has attracted considerable interest due to its technological simplicity and economic potential [13,14,15,16]. *Fast pyrolysis* (at moderate temperatures) generally leads to the thermal decomposition of organic materials in an oxygen-free or oxygen-limited atmosphere, yielding a variety of products of interest (gases, liquids, and some solid char products). Carbonization of organic matter (often reported as *slow pyrolysis*) is usually a process of incomplete thermal conversion (again in the absence of oxygen and most commonly at temperatures between 300 and 800 °C), mainly yielding a carbon-rich solid matter [22,23,24]. The process is primarily focused on biochar yield and its physicochemical properties, which strongly depend on the feedstock and the chosen reaction parameters that govern the aromaticity, carbon content, pore volume, and abundance of oxygen-containing functional groups in the carbon matrix [24,25,26,27,28,29]. Biochar produced via carbonization exhibits high porosity, surface area, and chemical stability [12,30,31]. From this perspective, the carbonization temperature and the heating rate are the most critical process parameters, providing control over the conversion efficiency, biochar yield, and products’ general characteristics, e.g., the ratio of fixed carbon (FC) versus volatile matter (VM) content, biochar enrichment with carbon (C), its morphological transformations, and others [32]. Cordero et al. [22] proposed an optimal temperature range between 300 and 700 (max. 800) °C. Ronsse et al. [23] reported that FC content between 60 and 80% for woody biochar is normally obtained at 400 to 600 °C. According to Ferreira et al. [24], the biochar derived for use as a catalyst, fertilizer, or for environmental applications is produced at 400 to 600 °C. The authors confirmed that at higher temperatures, the biochar has lower H/C and O/C ratios. This induces a higher degree of coalification, which is desired when biochar is utilized as fuel.

According to Monga et al. [33], the surface functionalities of biochars can be systematically engineered by altering the nature of the raw feedstock (lignocellulosic or non-lignocellulosic) or by introducing chemical modifiers such as metal ions, rare-earth elements, or surfactants. Biochar properties can be specified through numerous modification strategies (see, e.g., Fakhar et al. [34]). Well-known techniques for physicochemical activation of biochar include impregnation, chemical activation, precipitation, or metal oxide deposition [35,36,37]. Chemical activation with acids or bases (e.g., H_3_PO_4_, HCl, or KOH) typically enhances biochars’ porosity and introduces acidic or oxygen-containing functional groups [33]. It may also induce structural degradation and require extensive post-treatment, as discussed by Nanda et al. [38]. Similarly, precipitation-based and high-temperature metal oxide deposition methods often suffer from poor homogeneity [39], pore blockage [40], and high energy demand [41]. These limitations highlight the need for a modification approach capable of ensuring uniform metal incorporation while preserving the integrity of the carbon structure. Surface modification with metal ions, in particular Fe^2+^ or Zn^2+^, has been reported to favor the adsorption behavior [42,43], electrical properties [14,44], and surface chemistry of biochars [45,46]. Zinc is known for its comparatively low cost, toxicity, and regulatory acceptance in comparison to other commonly used ions for biochar modification, e.g., Cu^2+^, Ni^2+^, Co^2+^, and others [47,48], and this element is an essential micronutrient (see Plum et al. [49]). According to Sayed et al. [48], Zn^2+^-activated biochar demonstrates high chemical stability and reduced metal leaching over a broad pH range, outperforming Fe- and Cu-based analogues. The use of such materials shows sustainable utilization in environmental/biological sensing, water treatment, and gas purification/sensing (see Liu et al. [47]). Inoue [50] stated that solvothermal modification is a powerful method capable of enabling effective interaction between the metal precursors and the carbon matrix while controlling the distribution and morphology of metal ions. The solvothermal process provides a confined CO_2_—negative reaction environment [51], well implemented at different temperatures or even pressures [50], promoting uniform diffusion of the metal ions into the porous biochar structure [50,52] while reducing agglomeration risks [47,53,54].

Building on previous in-house [31,55] and independent [22,23,24,47,48,50,52,53,54] research, the present study aimed to transform lavender agro-industrial residue into a contemporary functional material through its carbonization at two temperatures (450 °C and 650 °C), followed by solvothermal Zn^2+^ modification using two concentrations (3 and 5 mmol). The effects of pyrolysis temperature and zinc incorporation on the chemical composition and morphological properties of solvothermally modified biochar particulates were examined. A moderate temperature dependence of biochar yield was confirmed, while X-ray Fluorescence (XRF) analysis showed a limited effect of carbonization temperature on the detectable elemental composition in the obtained materials. The combined effects of temperature (during feedstock carbonization and solvothermal modification) and Zn^2+^ incorporation on the biochar’s surface chemical characteristics, structure, and morphology were observed using Fourier Transform Infrared (FTIR) spectroscopy and Scanning Electron Microscopy/Energy-Dispersive X-ray Spectroscopy (SEM/EDS).

## 2. Results and Discussion

### 2.1. Effect of Carbonization Temperature on Biochar’s Yield and Chemical Composition

Biomass carbonization was carried out at two temperatures (450 and 650 °C), which were chosen to represent a moderate range relevant to practical considerations for biochar production. The current work was based on earlier in-house investigations, described in detail by Petrova et al. [55] and Naydenova et al. [31].

The temperature dependence of biochar yield was estimated according to the procedure described by Petrova et al. [55]. Thus, 32% of biochar was obtained at a carbonization temperature of 450 °C, and 28% was obtained at 650 °C. As expected, the results corresponded well with previous studies [24,31,55].

The elemental composition of lavender residues and their thermally converted derivatives was experimentally measured using the XRF technique. Table 1 provides a comparative overview of the data obtained, along with their estimated standard deviation (±SD). The largest temperature effect was observed when comparing the air-dried biomass with the biochar. Although moderate differences in the detected elemental composition were determined for the biochar obtained at two different temperatures, they reflected systematic and reproducible trends associated with progressive carbonization rather than complete structural transformation [22,24,55].

The oxygen content (O) showed a notable decrease in the carbonized products in contrast to the air-dried biomass. This result corresponds with the decomposition of oxygen-containing functional groups (–OH, C=O, COOH) during carbonization, as it was observed in previous investigations [31,55].

The present analysis showed a slight but observable temperature-dependent effect of enrichment for potassium (K), chlorine (Cl), sulfur (S), and iron (Fe) in contrast to the other identified elements. This suggests the retention of compounds such as sulfates and chlorides in the biochar structure. Fertilizers typically used for lavender farming usually contain essential macronutrients that stimulate plant cell growth, primarily nitrogen (N), phosphorus (P), and K, plus micronutrients such as Fe in relatively higher concentrations in comparison with other micronutrients [55,56,57]. A recent study by Peçanha et al. [56] discussed the effects of complementation with K-containing substances (such as KCl or K_2_SO_4_), often applied as a routine agricultural practice for increasing the yield and quality of essential oils. Vassilev et al. [16] discussed the higher thermal mobility and reactivity of K species in lignocellulosic biomass, which favors their preferential retention and accumulation in the ash fraction at elevated temperatures. Similar effects were observed and discussed elsewhere by Buss et al. [58]. Grafmüller et al. [59] suggested that such a trend can be attributed to the progressive thermal degradation of the organic matrix during slow pyrolysis, which leads to a relative concentration of inorganic constituents in the resulting biochar. The lack of temperature dependence in the experimentally measured concentration of the other mineral compounds in biochar (450 and 650 °C) suggests the presence of a stable mineral structure.

Traces of heavy metals (e.g., Pb) were found in negligible amounts and are not included in the comparative discussion. The inorganic composition of biomass and the derived biochar strongly depend on the biomass type, soil quality, applied agricultural procedures, carbonization conditions, ash content, and mineral profiles of different feedstocks and enrichment activities [58]. Buss et al. [58] discussed that enrichment of biochar with K and Ca has a significant impact on morphology and pore development, while Fe is often related to electrical conductivity and catalytic activity. The topic is considered also in [59,60,61,62]. A recent study by Grafmüller et al. [63] used wood ash additives in biomass pyrolysis and proposed an approach for enhanced nutrient recycling and carbon sequestration in soil. The obtained XRF data were consistent with the results reported elsewhere [47,53,54,55,60,61,62]. However, the influence of the inorganic content is beyond the scope of the present study.

### 2.2. Effect of Carbonization Temperature and Solvothermal Zn^2+^ Modification on Biochar’s Chemical Reactivity

**Figure 1:**
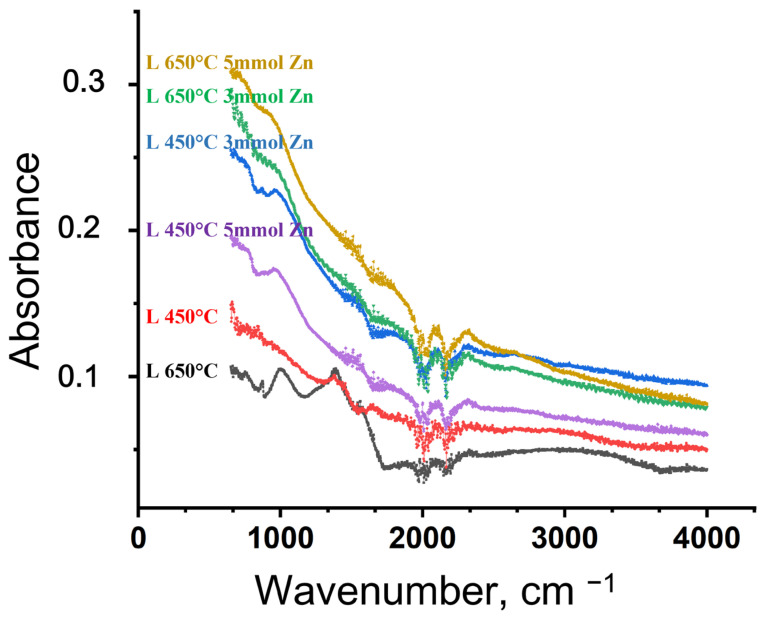
FTIR spectra of biochars obtained from lavender (L) biomass at 450 °C and 650 °C, and their Zn^2+^-modified derivatives at two concentrations (3 mmol and 5 mmol).

FTIR spectroscopy was used to determine the main functional groups present on the surface of biochar obtained from lavender residues at temperatures of 450 °C and 650 °C, both before and after its solvothermal modification with Zn^2+^ (see Figure 1). The numerical values for the peak positions, intensities, and areas in the spectra of the biochar samples were obtained by digitally processing the original FTIR data, recorded in the range 400–4000 cm^−1^. The data were exported and processed in OriginPro 2024, and for each identified peak, the main parameters—position, height, and integral area—were determined and used for comparative analysis of the different samples.

The FTIR spectra of the biochar samples produced at 450 and 650 °C, as well as those modified with zinc, exhibited characteristic absorption bands typically observed in lignocellulosic-derived carbon materials. In line with the findings reported by Naydenova et al. [31], the broad band in the 3200–3600 cm^−1^ region was attributed to the O–H stretching vibrations of hydroxyl groups and the adsorbed moisture. With increasing pyrolysis temperature, a gradual decrease in the intensity of the O–H, C=O (1700–1600 cm^−1^), and C–O (1000–1300 cm^−1^) bands was observed, indicating progressive carbonization and loss of oxygen-containing functional groups. The results correspond well to those presented by Li et al. [64] and Wang et al. [65].

For the Zn-modified samples, the attenuation and broadening of absorption features in the low-wavenumber region (600–700 cm^−1^) were observed, which are commonly associated with the presence of metal–oxygen interactions or inorganic Zn-containing species on the biochar surface [66,67]. Although this spectral region is subject to band overlap and does not allow unambiguous identification of specific Zn–O bonding configurations, the observed changes were consistent with the successful incorporation of Zn species, as further supported by SEM/EDS elemental analysis.

### 2.3. Effect of Carbonization Temperature and Solvothermal Zn^2+^ Modification on Biochar’s Structure and Morphology

**Figure 2:**
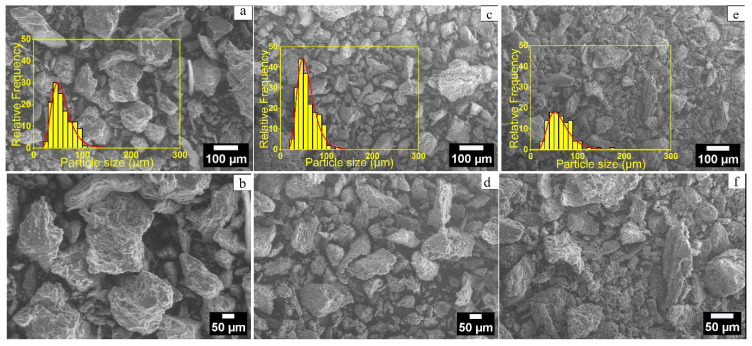
SEM images of lavender biomass and its carbonized derivatives acquired at 50 µm and 5 µm scales: (**a**,**b**) air-dried lavender biomass residue; (**c**,**d**) lavender biochar obtained at 450 °C; (**e**,**f**) lavender biochar obtained at 650 °C.

**Figure 3:**
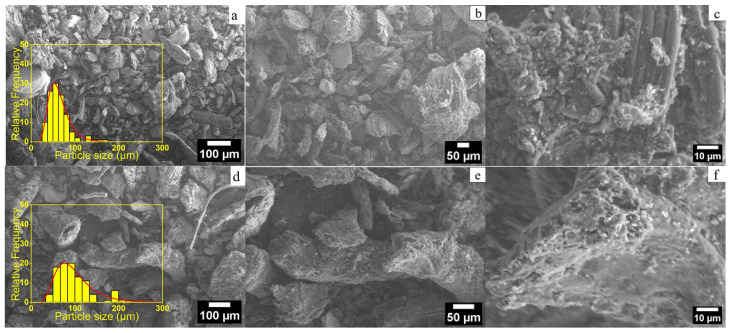

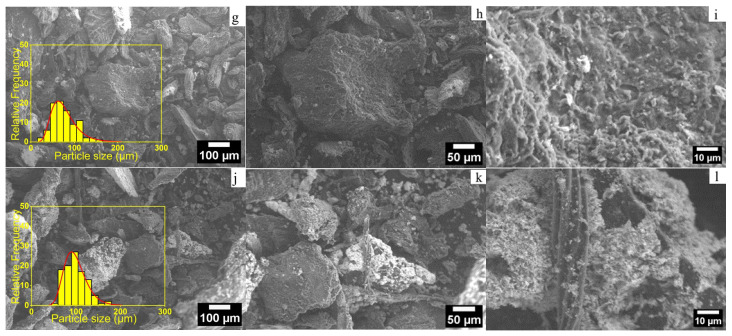
SEM images of Zn-modified lavender biochar acquired at 100 µm, 50 µm, and 5 µm scales: (**a**–**c**) biochar carbonized at 450 °C, modified with 3 mmol Zn^2+^; (**d**–**f**) biochar carbonized at 450 °C, modified with 5 mmol Zn^2+^; (**g**–**i**) biochar carbonized at 650 °C, modified with 3 mmol Zn^2+^; (**j**–**l**) biochar carbonized at 650 °C, modified with 5 mmol Zn^2+^.

The data obtained through SEM/EDS analysis allowed for a comparative assessment of the structural and morphological transformations occurring in the biochar particulates as direct effects of the biomass treatment procedures. Within the scope of the analyses conducted, the samples were prepared according to the methodologies described in Section 3.2. Herein, Figure 2 presents the SEM images of the air-dried solid biomass and the biochar derived at 450 and 650 °C, whereas Figure 3 shows the Zn-modified derivatives of the biochar using solutions with two different Zn^2+^ concentrations (3 and 5 mmol).

Particle mean size data, as presented in Table 2, were calculated from the SEM images. For this purpose, ImageJ software (version 1.54g; National Institutes of Health, Bethesda, MD, USA) was used. Particle boundaries were manually outlined, and the Feret diameter was measured for at least 100 particles per sample to ensure statistical reliability. The mean particle size and standard deviation (SD) were then calculated.

The SEM analysis allowed for estimating the combined effects of carbonization temperature and Zn^2+^ modification on biochar’s structure and morphology. As shown in Table 2 and Figure 2, the lavender biomass exhibited an average particle size of 67.2 ± 3.8 µm (Figure 2a,b). Carbonization at 450 °C (Figure 2c,d) reduced the particle size to 60.4 ± 1.9 µm. Increasing the carbonization temperature to 650 °C (Figure 2e,f) resulted in a further slight decrease in the biochar’s particle mean diameter (58.4 ± 1.9 µm), supporting the hypothesis that elevated temperatures lead to more compact and stable carbon structures due to intensified organic matter degradation and carbon condensation [68].

Moderate Zn^2+^ modification (3 mmol, Table 2 and Figure 3a–c) of the samples carbonized at 450 °C allowed achieving uniform Zn incorporation without significant agglomeration, maintaining a stable porous structure, whereas excessive Zn^2+^ modification (5 mmol) promoted particle clustering, potentially limiting pore accessibility and reducing the effective surface area, as shown in Figure 3d–f. Similarly, the biochar carbonized at 650 °C and modified with 3 mmol Zn^2+^ (Figure 3g–i) yielded a moderate particle size of 76.7 ± 2.9 µm, whereas modification with 5 mmol Zn^2+^ (Figure 3j,k) led to an average particle size of 99.2 ± 2.5 µm. Overall, the combination of carbonization at 650 °C with 3 mmol Zn^2+^ (Figure 3g–l) appeared to be optimal for the lavender biochar, providing a well-developed carbonaceous surface, controlled Zn^2+^ incorporation, and reasonable mean particle size, all of which have been identified as critical factors for structural stability and morphological uniformity [22,23]. These results are consistent with previous investigations that confirmed that higher carbonization temperatures yield smaller particles and more stable biochar structures, whereas Zn modification enhances the material’s sensing properties [68,69,70].

**Figure 4:**
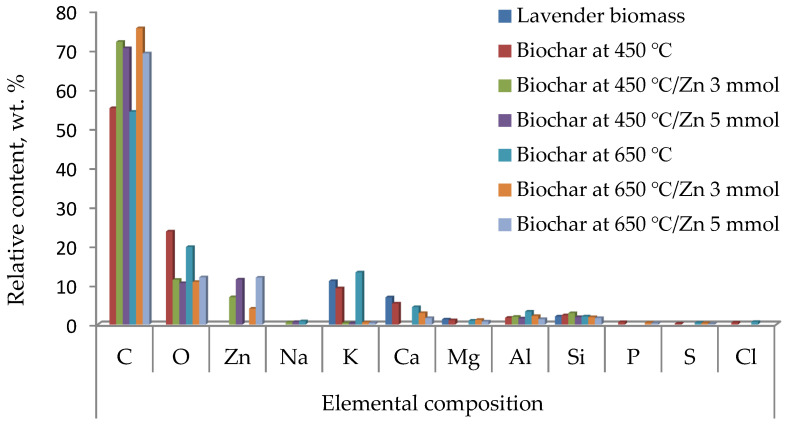
Elemental compositions determined by EDS analysis of lavender biomass, biochar obtained at 450 °C and 650 °C, and Zn-modified derivatives.

Figure 4 illustrates the samples’ chemical compositions, which were estimated by EDS analysis for lavender biomass, biochar (at 450 and 650 °C), and its Zn-modified derivatives (with 3 and 5 mmol of Zn^2+^).

Besides its limited representativeness, the EDS analysis was consistent with the trends observed in the experimentally measured characteristics of the lavender biochar using the above-described analytical methods and techniques.

Although the present study was limited to describe the preparation of Zn-modified biochar and its general characterization, a large number of practical applications of similar products have been investigated. Modified biochar shows distinct ecological significance. The need for targeting particular problems necessitates the production of biochar with specific compositions and properties [61,68,70,71,72,73,74,75,76]. Metal-modified biochars with comparable composition, structural characteristics, and surface chemistry (similar to those determined in the present work) have been reported in the literature as promising platforms for sensing and/or electrochemical [61,73,74], catalytic [70,72,75,76], and environmental applications [62,77,78,79]. Wang et al. [13] studied the electrochemical supercapacitor performance of modified lavender biochar. Zhang et al. [77] reported the potential application of Fe/Zn-modified biochar in purifying tap water or wastewater contaminated by microplastics. A recent review by Gusiatin and Rouhani [78] described the application of selected methods for biochar modification (aiming to adjust its functionalization) for the immobilization of metal ions in contaminated soil. Liu et al. [47] proposed Fe-Zn-modified sludge-derived biochar for adsorption of herbicide in aqueous solution. Yan et al. [79] obtained high adsorption performance for tetracycline using Zn-modified biochar, derived from aerobic granular sludge.

## 3. Materials and Methods

### 3.1. Biomass Origin and Characterization

The essential oil and agro-industrial sectors generate significant amounts of biomass residues, which are often discarded as low-value waste. In the present work, lavender residue, collected as a waste product from essential oil production, was used as feedstock for biochar production. The biomass was characterized through proximate, ultimate, ash, lignocellulosic, and calorimetric analyses. The raw material was ground and sieved (through a sieve with a pore diameter <1 mm), and all samples were prepared in accordance with ISO 16559:2022 [80] and ISO 14780:2017 [81]. The proximate analysis of the air-dried biomass showed the samples’ weight percentages (wt.%) of moisture, volatile matter, and ash according to ISO 18123:2023 [82], ISO 18122:2022 [83], and ISO 18134-3:2023 [84]. Standard procedures were applied [55] to estimate the FC fraction by the difference: FC = 1 (Wt + A + V). In this equation, Wt is the total relative moisture content, and A and V are the relative ash and volatile matter contents, respectively. The ultimate analysis was performed using an Elemental Analyzer EuroVector EA 3000 (EuroVector S.p.A., Milan, Italy), whereas an Inductively Coupled Plasma Optical Emission Spectroscopy (ICP-OES) was used for the ash analysis (see [31]). The obtained results (summarized in Table 3) were found to be consistent with previous in-house [31,55] and independent [16] studies.

### 3.2. Biochar Preparation and Modification

**Figure 5:**
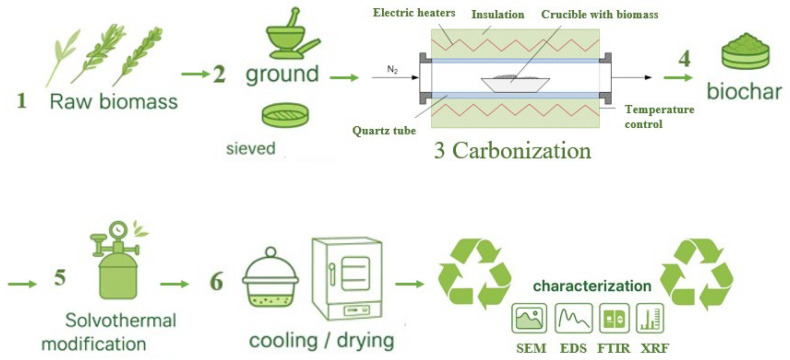
Process flow diagram.

**Figure 6:**
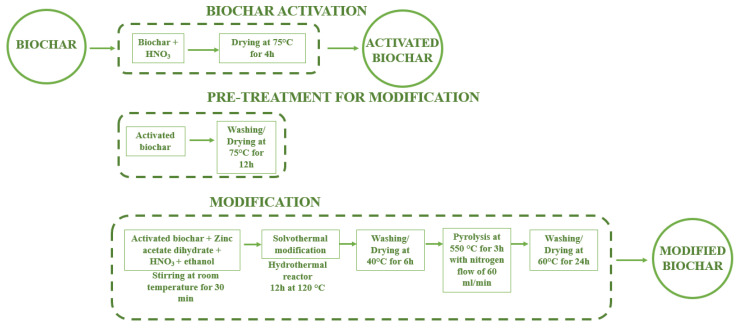
Solvothermal modification diagram.

The biomass carbonization was processed in an in-houselaboratory-scale horizontal tube furnace (HTF), in accordance with previously described methods and procedures [24,31,55]. The biochar was obtained under a nitrogen atmosphere at atmospheric pressure, using a constant N_2_ flow rate of 1 L/min and a residence time of 3 h in the reaction zone. In the present study, two carbonization temperatures were applied (450 °C and 650 °C), in line with previous studies demonstrating an expected T-dependence of the char properties [22,23,24]. A schematic overview of the overall experimental workflow is presented in Figure 5, while Figure 6 illustrates the solvothermal Zn^2+^ modification in detail.

The preparation of Zn-modified biochar involved two main stages: (i) chemical pre-activation of biochar, and (ii) solvothermal incorporation of Zn^2+^ ions. Prior to Zn^2+^ modification, all biochar samples were uniformly treated with 1 M HNO_3_ in order to activate the surface and ensure comparable initial conditions. However, the effect of this treatment was not considered an independent variable in the subsequent analysis. During the pre-activation step, approximately 20 g of biochar was treated with 200 mL of 1 M HNO_3_ at 75 °C for 4 h to introduce oxygen-containing surface functionalities. The samples were then washed with deionized water until neutral pH.

The solvothermal modification of carbonized biomass was the novel part of the current investigation. It was conducted in a 35 mL Teflon-lined stainless-steel autoclave using ethanol as a solvent. The pre-activated biochar was dispersed in a nitric acid–ethanol mixture and stirred at room temperature for 30 min to obtain a homogeneous suspension. Zinc was introduced in the form of zinc acetate dihydrate (Zn(CH_3_COO)_2_·2H_2_O), which served as the Zn^2+^ precursor. The amount of precursor was calculated to obtain a total Zn^2+^ content of either 3 or 5 mmol in a total suspension volume of 30 mL. The resulting suspension was subjected to solvothermal treatment at 120 °C for 12 h. After cooling to room temperature, the product was thoroughly washed with deionized water to remove residual ions and dried at 40 °C for 6 h.

A subsequent thermal treatment was applied at 550 °C for 3 h under a nitrogen atmosphere (N_2_ flow rate: 60 mL/min; heating rate: 30 °C/min), following the procedure reported in [19], to stabilize the Zn^2+^ biochar surface. The final material was washed again with deionized water and dried at 60 °C for 24 h. In this paper, the obtained samples were referred to as Zn-modified biochars.

All chemicals used in this study, including HNO_3_, Zn(CH_3_COO)_2_·2H_2_O, and ethanol, were of analytical grade and used without further purification.

### 3.3. Characterization of the Products Using XRF, FTIR, and SEM/EDS Analyses

The obtained materials were examined by XRF analysis to determine the elemental composition of the starting material (mainly inorganic elements), using an E-lite XRF analyzer (Z-Spec, East Greenbush, NY, USA).

The functional groups (e.g., OH, C=O, C–O, C=C, Zn–O) were identified using FTIR spectroscopy. The spectra were measured using a Cary 630 spectrometer (Agilent Technologies Inc., Santa Clara, CA, USA) equipped with a Diamond-ATR (Attenuated Total Reflectance) accessory.

In addition, SEM analysis was carried out using the SEM-Zeiss EVO 10 apparatus, manufactured by Carl Zeiss Microscopy GmbH, Carl-Zeiss-Promenade 10, 07745 Jena, Germany. It was used to analyze the particulate structure and morphology, whereas the elemental composition was analyzed using an EDS detector, namely an Oxford Instruments EDS Xplore 30, manufactured by Oxford Instruments, Halifax Rd, UK.

In the present study, ChatGPT (GPT-4, OpenAI, San Francisco, CA, USA) was used solely to improve some parts of the English language and for grammatical corrections. All edits provided by the tool were reviewed and verified by the authors, who take full responsibility for the final content.

## 4. Conclusions

The present study proposed an experimental investigation aiming to utilize solid lavender agro-waste from the essential oil industry through carbonization and solvothermal modification. The solvothermal transformation of biochar (which is processed in a solvent at elevated pressure and temperature) is an effective approach for biomass modification, allowing for the production of structurally tailored carbon-based materials that are broadly applicable, inexpensive, and environmentally friendly.

The products’ characterization showed a limited influence of carbonization temperature on biochar yield and elemental composition. The observed trends indicated progressive carbonization rather than a complete structural transformation of the biomass feedstock. The combined effects of thermal decomposition and Zn^2+^ incorporation influenced the structural characteristics and surface chemistry of the obtained materials, as confirmed by morphological and elemental analyses. Comparative evaluation showed that moderate Zn^2+^ loading (3 mmol) of biochar obtained at 650 °C resulted in more homogeneous surface morphology and improved structural stability, in contrast to the unmodified samples and those treated with higher Zn concentrations. Thus, the combination of elevated carbonization temperature and modification with a low concentration of metal ions provided a good balance between reduced carbon and oxygen content, particle structure, size, and porosity, as well as homogeneous metal incorporation, resulting in improved structural stability of the obtained materials, which typically improves particles’ conductivity, surface stability, activity, and sensitivity [22,23,61,66,68,70,73,74].

The present work proposed a moderate approach for the utilization of agro-industrial residues, combining two well-known methods, thus providing a well-defined structural basis for future studies, with a focus on the functional performance evaluation and application-oriented testing of the derived products.

## Figures and Tables

**Table 1:** Elemental composition (obtained through XRF analysis) of lavender biomass residue before and after carbonization at 450 °C and 650 °C under atmospheric conditions.

Element	Lavender Residue		Carbonized Lavender at 450 °C		Carbonized Lavender at 650 °C	
	Value ± SD	Unit	Value ± SD	Unit	Value ± SD	Unit
**O**	87 ± 0.1	%	60 ± 0.1	%	58 ± 0.3	%
**Na**	2 ± 0.1	%	3 ± 0.3	%	3 ± 0.5	%
**K**	4 ± 0.01	%	10 ± 0.02	%	16 ± 4.8	%
**Ca**	2 ± 0.006	%	6 ± 0.02	%	6 ± 0.01	%
**Si**	2 ± 0.01	%	4 ± 0.01	%	4 ± 0.002	%
**Mg**	9061 ± 0.4	ppm	2 ± 0.1	%	2 ± 0.04	%
**Al**	4652 ± 5.3	ppm	1 ± 0.02	%	1 ± 0.01	%
**P**	3192 ± 3.2	ppm	7916 ± 2.3	ppm	7816 ± 0.5	ppm
**S**	2349 ± 0.8	ppm	2046 ± 4.8	ppm	2103 ± 1.9	ppm
**Cl**	2632 ± 1.7	ppm	4644 ± 4.8	ppm	5357 ± 8.6	ppm
**Fe**	8020 ± 5.1	ppm	231,578 ± 19.43	ppm	24,208 ± 2.6	ppm

**Table 2:** Average particle size estimated for air-dried solid lavender biomass particulates before and after carbonization (450 °C and 650 °C) and Zn^2+^ modification.

Biomass Sample	Particle Size * ± SD (µm)
Lavender (air-dried solid biomass residue)	67.2 ± 3.8
Biochar obtained at 450 °C	60.4 ± 1.9
Biochar obtained at 650 °C	58.4 ± 1.9
Biochar obtained at 450 °C/Zn^2+^ 3 mmol	66.1 ± 2.6
Biochar obtained at 450 °C/Zn^2+^ 5 mmol	99.1 ± 4.4
Biochar obtained at 650 °C/Zn^2+^ 3 mmol	76.7 ± 2.9
Biochar obtained at 650 °C/Zn ^2+^ 5 mmol	99.2 ± 2.5

* Estimated from the SEM images.

**Table 3:** Thermo-chemical characteristics of the lavender residue used.

Proximate Analysis ^2^, wt. %		Ash Analysis (wt. %, db)	
Volatiles, db	68.34	SiO_2_	8.56
Fixed carbon, db ^1^	11.15	Al_2_O_3_	2.60
Moisture, r	7.15	Fe_2_O_3_	1.23
Ash, db	13.36	MnO	0.08
Ultimate analysis ^2^, wt. %, db		CaO	14.67
C	41.14	MgO	3.72
H	6.17	BaO	0.07
N	1.80	Na_2_O	0.66
S	2.01	K_2_O	9.50
O ^1^	28.27	Cr_2_O_3_	0.01
C/H	0.56	TiO_2_	0.26
C/O	1.94	ZnO	0.02
HHV ^2^, db, MJ/kg	17.45	CuO	0.01
Lignocellulosic analysis ^2^, wt. %, db		SrO	0.06
Cellulose	28.60	P_2_O_5_	1.53
Lignin	31.70	Co_3_O_4_	<0.01
Pentosans	13.10	PbO	0.01
Extractives	3.05	CdO	<0.01
		As_2_O_5_	<0.01
		Losses on ignition (LOI)	35.22

^1^ By difference; ^2^ Reported elsewhere [85]; Abbreviations: db—on a dry basis; r—as received.

## Data Availability

The original data presented in this study are included in the article. Any further inquiries should be directed to the corresponding authors.
